# Midgut malrotation presenting with hyperemesis gravidarum: A case report

**DOI:** 10.1097/MD.0000000000029670

**Published:** 2022-07-29

**Authors:** Hongjiang Zhao, Linzhen Wu, Bin Yang, Hongkai Shang

**Affiliations:** a The Fourth School of Clinical Medicine, Zhejiang Chinese Medical University, Hangzhou, China; b Department of Gynecology, Affiliated Hangzhou First People’s Hospital, Zhejiang University School of Medicine, Hangzhou, China; c Department of Obstetrics, Affiliated Hangzhou First People’s Hospital, Zhejiang University School of Medicine, Hangzhou, China; d Department of Radiology, Affiliated Hangzhou First People’s Hospital, Zhejiang University School of Medicine, Hangzhou, China.

**Keywords:** case report, midgut malrotation, pregnancy

## Abstract

**Rationale:**

Midgut malrotation is a rare congenital abnormality resulting from failure of complete intestinal rotation and subsequent fixation during early fetal development. There appeared to be no obvious symptoms in most patients, and a few patients may exhibit symptoms similar to hyperemesis gravidarum, such as nausea and vomiting. Here, we present a case of midgut malrotation presenting as hyperemesis gravidarum.

**Patient concerns:**

A 27-year-old woman with an intrauterine pregnancy of 27 + 6 weeks complained of severe nausea and vomiting for 2 weeks.

**Diagnosis:**

Magnetic resonance imaging showed obvious dilatation in the proximal part of the duodenum and gastric cavity and the absence of a duodenal path dorsal to the superior mesenteric artery, which was diagnosed as midgut malrotation.

**Interventions:**

Considering that the patient’s vital signs were stable, without manifestation of peritonitis or the risks of surgery to the fetus, conservative treatment was adopted. Unfortunately, the fetus developed severe hydrocephalus at 32 weeks. The patient and her family decided to abandon the fetus, and a mid-trimester-induced abortion was performed.

**Outcomes:**

The related symptoms completely disappeared after delivery, and the relevant examination after discharge also confirmed the presence of midgut malrotation without gastrointestinal discomfort within 1 year after delivery.

**Lessons:**

Midgut malrotation can be considered as a differential diagnosis of hyperemesis gravidarum. Conservative treatment under close monitoring is desirable in pregnant women diagnosed with midgut malrotation.

## 1. Introduction

Midgut malrotation is a congenital abnormality that develops from partial or complete failure of the midgut to rotate 270° counterclockwise around the axis of the superior mesenteric artery (SMA) during embryonic development,^[1]^ which leads to abnormal positioning and fixation of the small and large intestine. This condition typically presents in children^[2]^ and rarely in adults, with an estimated incidence of 0.2% to 0.5%, excluding pregnancy.^[3]^ At present, its etiology remains unclear, which is possibly related to mutations in forkhead box transcription factor (*FOXF1*) and mutations in the gene *BCL6* and *L–R* asymmetry genes.^[4–6]^ Most patients have no obvious symptoms, and only a few may present with intermittent abdominal pain, nausea, and vomiting.^[7]^

During pregnancy, up to 90% of women experience nausea and/or vomiting, and those with severe nausea and vomiting may have hyperemesis gravidarum (HG), which is associated with fluid, electrolyte, acid–base imbalance, nutritional deficiency, and weight loss.^[8]^ Although it generally occurs in the 4th to 10th week of pregnancy and resolves by mid-gestation, 15% to 20% of women will continue this symptom until the third trimester of pregnancy, even if 5% will maintain it until delivery.^[9]^

As midgut malrotation rarely occurs, inadequate knowledge of the disease and nonspecific symptoms may delay diagnosis or treatment, thereby increasing the incidence and risk of death. Currently, there are few reports of midgut malrotation discovered during pregnancy, most of which are treated by surgery. Here, we report a case of midgut malrotation with HG. As the patient’s condition was stable, conservative treatment was adopted, and the symptoms disappeared postpartum. We absorbed some experience from adverse fetal outcome events. This case can provide some lessons for clinicians and help enhance their awareness of these conditions.

## 2. Case report

A 27-year-old woman with a pregnancy of 27 + 6 weeks was hospitalized for severe nausea and vomiting, who began vomiting 2 weeks ago, about 4 to 5 times a day, denying fever, diarrhea, hematochezia, and melena. The results of medical examination in a local hospital indicated that the urine ketone body was examined to be +++, and blood potassium was 2.68 mmol/L, diagnosed with acute gastroenteritis and received anti-infection and rehydration treatment. The treatment lasting 10 days produced little effect, so she was transferred to our hospital. Our first consideration was the presence of HG.

**Figure 1.**
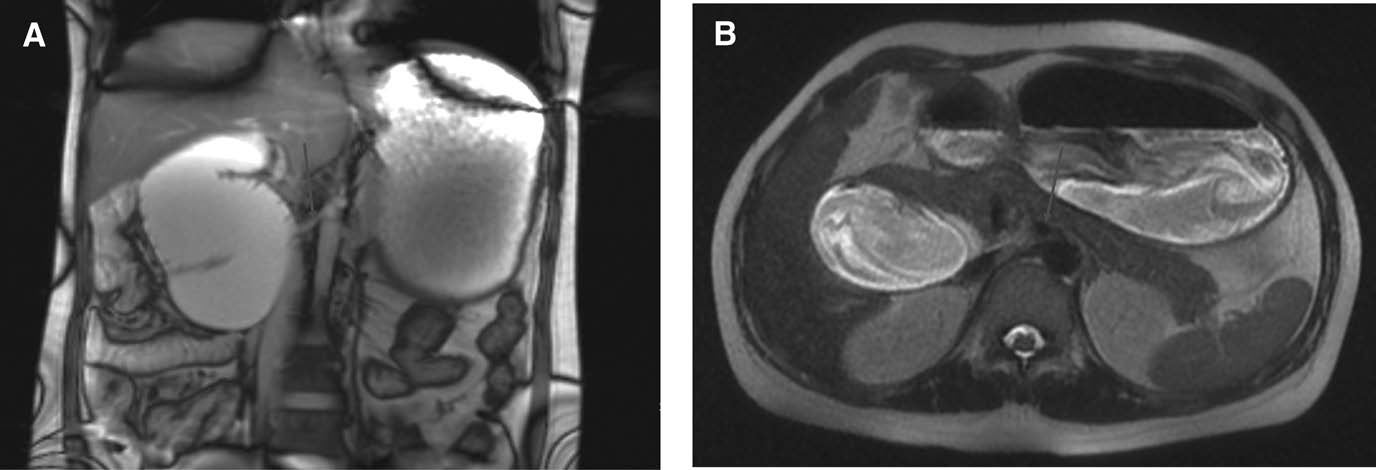
MRI image. (A). Coronal image showing the absence of a duodenal path dorsal to the SMA (purple arrow). (B) Axial image showing obvious dilation in the proximal part of duodenum and gastric cavity, and the absence of a duodenal path dorsal to the SMA (purple arrow). MRI = magnetic resonance imaging, SMA = superior mesenteric artery.

We have completed the relevant inspection, where the serial blood tests revealed metabolic alkalosis (pH 7.674, Base excess (BE) of 23 mmol/L), elevated lactate of 6.9 mmol/L, Alanine aminotransferase (ALT) of 1076 U/L, Aspartate aminotransferase (AST) of 584 U/L, K+ of 3.31 mmol/L, Cl– of 66 mmol/L, and evidence of acute kidney injury (creatinine: 294 mmol/L). The patient was immediately transferred to the intensive care unit for treatment, considering her critical condition, without abnormalities on digestive system ultrasound and electrocardiogram. Magnesium isoglycyrrhizinate (200 mg Quaque die (QD)) and acetylcysteine (8 g QD) were applied to protect the liver, fasting gastrointestinal decompression to alleviate vomiting symptoms, and fluid supplementation to manipulate electrolyte and acid–base imbalance. The patient underwent small intestinal magnetic resonance imaging (MRI) to eliminate nausea and vomiting caused by gastrointestinal disease, displaying a narrow horizontal segment of the duodenum, absence of a duodenal path dorsal to the SMA, significant dilation of the proximal duodenum and gastric cavity, and a large amount of chyme in the cavity (Fig. 1). We conducted a multidisciplinary discussion. Considering that the patient’s vital signs were stable, without manifestation of peritonitis and the risks of surgery to her fetus, conservative treatment was decided to be adopted, involving continued fasting, gastrointestinal decompression, establishment of intravenous nutritional support, and application of dexamethasone to promote fetal lung maturation. Subsequently, the patient’s indicators gradually returned to normal, and the symptoms were alleviated. The gastric tube was then removed, and a liquid diet was offered, while the patient vomited again with the liver function increasing further; thus, gastrointestinal decompression was continued. No abnormality was found on fetal ultrasound during regular examination, and the size of the uterus was consistent with the fetal age. Unfortunately, severe hydrocephalus in the fetus was found on ultrasound examination at 32 weeks of gestation. The patient and her family decided to abandon the fetus, and a mid-trimester-induced abortion was conducted.

**Figure 2.**
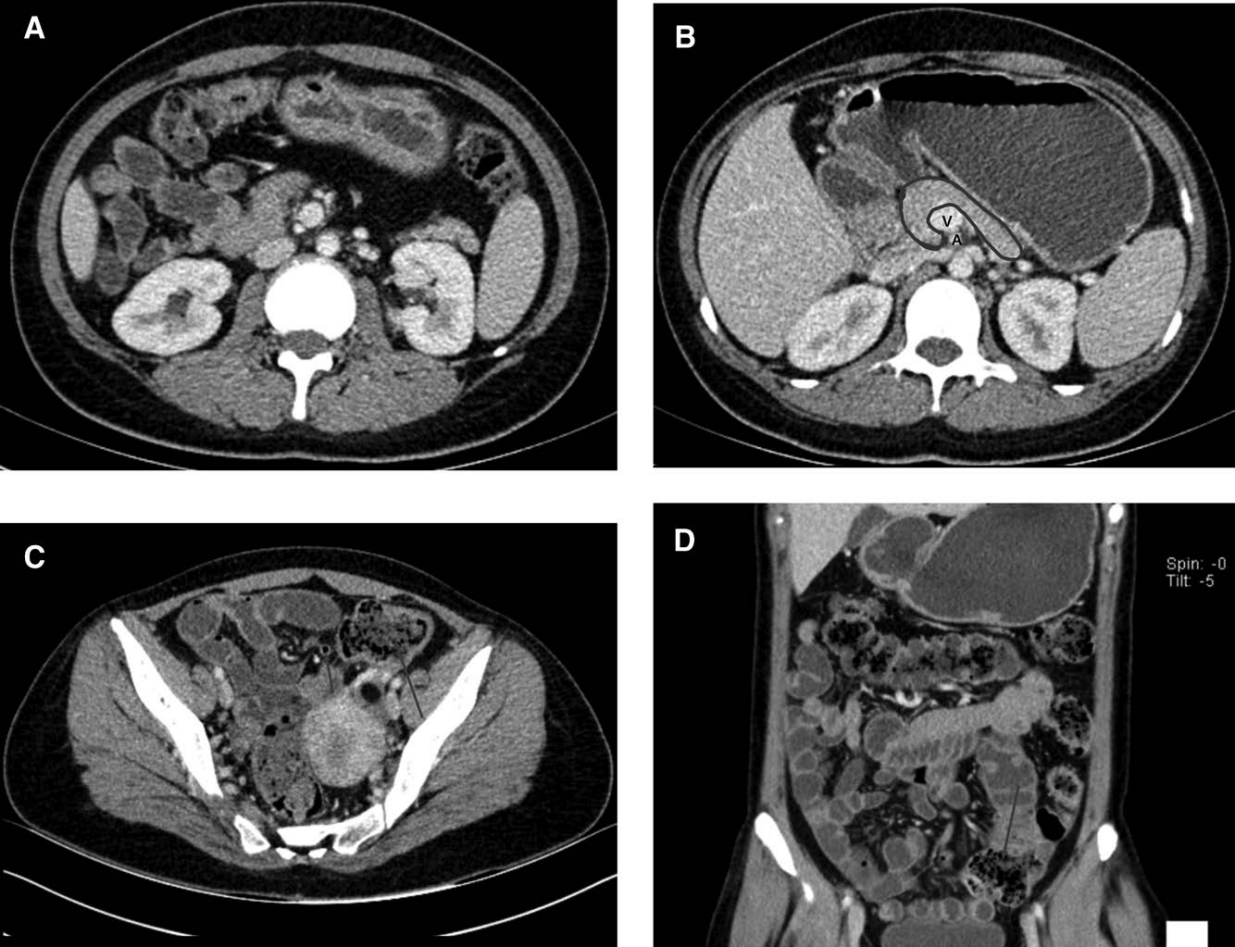
CT image. (A). Axial image shows the absence of a duodenal path dorsal to the SMA. (B) The shape of the pancreas is demonstrated outlined in purple. Note that the uncinate process is hypoplastic (a small amount of pancreatic tissue was seen posterior to the SMV, which did not extend beyond the left lateral margin of the SMV) in this case and that there is mesenteric vascular inversion with the SMV lying anterior to the SMA. (C) Axial image shows a left side of the midline positioned appendix (short arrow) and the cecum in the left lower abdomen (long arrow). (D) Coronal image shows small bowel on right and colon predominately on left. Note the cecum in the left lower abdomen (arrow). CT = computed tomography, SMA = superior mesenteric artery, SMV = superior mesenteric vein.

The symptoms of nausea and vomiting disappeared after the delivery. We removed the patient’s gastric tube and provided her with a liquid diet; no obvious nausea or vomiting occurred. After discharge, the patient underwent a relevant examination at another hospital, and the results still showed midgut malrotation (Fig. 2). There was no gastrointestinal discomfort within 1 year of delivery.

## 3. Discussion

Midgut malrotation is a rare disease with atypical clinical manifestations, where a few can show severe nausea and vomiting similar to HG, which is a diagnosis of exclusion. Alternative diagnoses should be considered when nausea and/or vomiting occurs after 9 weeks of pregnancy.^[8]^ Therefore, we are supposed to complete relevant examinations and tests to determine its severity and help control it on the one hand and exclude nausea and vomiting induced by other causes.^[8]^

As mentioned previously, midgut malrotation results from failure of complete intestinal rotation and subsequent fixation during early fetal development. Generally, midgut lengthening and rotation develop in gestational week 5, with the small intestine starting out as a straight tube and blood derived primarily from the SMA.^[10]^ Between weeks 5 and 10 to 11, the gut grows and herniates through the umbilical cord with the midgut loop rotating 90° anticlockwise around the axis of the SMA, bringing the duodenojejunal loop to the right and the colonic loop to the left side. In the 10th week, the intestines return to the abdominal cavity; the proximal loop of the bowel enters first, with the distal loop following up. Both the proximal and distal loops underwent a further 180° of anticlockwise rotation, with a total of 270° of rotation. The duodenojejunal loop lies posterior to and the cecocolic loop anterior to the SMA. The duodenojejunal junction lies in the left upper quadrant of the abdomen, and the ileocecal junction lies in the right lower quadrant.^[11]^ The clinical significance of malrotation abnormalities lies within 2 key features: an abnormally positioned duodenojejunal loop compressed by peritoneal Ladd bands eliciting obstruction of the duodenum and narrow fixation of the base of the mesentery placing the SMA at risk of volvulus.^[12,13]^

The patient was asymptomatic when not pregnant, while nausea and vomiting occurred during pregnancy, which may be due to abnormal duodenal torsion or folding with enlargement of the uterus, resulting in duodenal obstruction.

Considering the nonspecific symptoms, laboratory examinations, and the diagnosis of midgut malrotation mainly depend on plain abdominal radiography, upper gastrointestinal radiography, barium enema, computed tomography (CT), and MRI.^[14]^ Conventional radiography is neither sensitive nor specific for malrotation, despite the possible suggestion of a right jejunal marker and colon without stool filling in the right lower abdomen. The barium meal series of the upper gastrointestinal tract remains accurate; that is, the duodenal jejunal junction does not cross the midline and lies below the level of the duodenal bulb.^[7]^ CT imaging evaluation involves the right small intestine and left colon, which may be ignored. Signs of an inverse correlation between the superior mesenteric vessels, aplasia, or hypoplasia of the uncinate process and the absence of a duodenal path dorsal to the SMA are easier to identify,^[15,16]^ while the hesitation to use radiology during pregnancy is a major obstacle to diagnosis, mainly originating from the fear of radiation to the fetus.^[17]^ Ultrasound and MRI have proven to be safe without X-ray examination.^[18]^ However, they may fail to detect poor rotation, as the growing uterus fills most of the space in the abdominal cavity, inducing intestinal displacement, which masks poor rotation, especially among inexperienced radiologists. These factors make diagnosing rotation during pregnancy difficult.

On imaging, the following abnormalities were found: first, abnormal position of intestine and no duodenal path exists from the dorsal side to the SMA (Figs. 1 and 2A). The small intestine was located on the right, colon on the left, and cecum in the left lower abdomen (Fig. 2C, D). Second, abnormalities of the mesenteric vessels (Fig. 2B). The deviation from the normal relationship between the SMA and superior mesenteric vein (SMV) is a weighted indicator of malrotation,^[19]^ where the SMV is normally located on the right side of the SMA, vertically in front of the SMA, and inversely on the left side of the SMA. In most patients with malrotation, the SMA and SMV assume a vertical relationship or inverted inversion.^[16]^ However, the abnormal position of the SMV is not completely diagnostic, as some patients with malrotation may have a normal positional relationship, while patients without malrotation may also have a vertical or inverted relationship.^[20]^ Therefore, when CT shows an abnormal relationship of the mesenteric vessels, it is necessary to carefully evaluate whether there is an abnormal intestinal position. Third, the uncinate process was hypoplastic (Fig. 2B). Aplasia of the pancreatic uncinate process has been recognized as an indicator of midgut malrotation,^[21]^ which may be due to the incompletely rotated ventral bud of the pancreatic primordium, accompanied by midgut malrotation.^[22]^ Inoue and Nakamura described aplasia or hypoplasia of the uncinate process in a small series of 5 patients with malrotation, with no malrotation of a normal uncinate process in 101 patients. Chandra et al^[23]^ described aplasia and hypoplasia as the most common variations of pancreatic contour in patients with complete malrotation, with a total of 86% (18/21) of cases exhibiting uncinate process anomalies: 9 cases of aplasia and 9 cases of hypoplasia. A correct diagnosis can be made when a typical abnormal intestinal position, extraintestinal abnormalities, and abnormal orientation of SMV–SMA are determined.

Currently, there are no consistent guidelines for the treatment of midgut malrotation. Midgut malrotation may induce various life-threatening acute abdominal diseases, involving volvulus, paraduodenal hernia, and gastroduodenal intussusception.^[3,21,24]^ Most reports of midgut malrotation during pregnancy are complicated by acute abdominal diseases; therefore, surgical treatment is required, and reports on the treatment of patients with stable disease are lacking. Yin et al^[25]^ proposed that the treatment method is consistent with that of intestinal obstruction; if complications such as intestinal volvulus, ischemia, and intestinal necrosis occur, malrotation can be treated conservatively, involving gastrointestinal decompression, parenteral nutrition, and antispasmodic drugs. Zhao et al proposed that in emergency situations, emergency surgery should be adopted to relieve obstruction or volvulus, treat complications, and normalize the anatomy. Before 28 weeks of pregnancy, we should try to resolve the obstruction while maintaining the pregnancy. After 32 weeks of pregnancy, digestive surgery must be performed after emergency cesarean section. Between 28 and 32 weeks of pregnancy, the termination of pregnancy should be decided based on the development of the fetus and the situation of the pregnant women.^[26]^ Ladd procedure is a standard surgical treatment, including counterclockwise detorsion of the bowel, division of anomalous peritoneal fibrous bands (Ladd bands), broadening of the mesenteric base, appendectomy, and repositioning of the small bowel and cecum to the right and the large bowel to the left of the abdominal cavity.^[24]^ Gut malrotation correction surgery “Kareem procedure,” a new surgical procedure, is offered with completion of the 270° embryonic counterclockwise rotation, reversal of vascular inversion, and fixation of mesenteric attachments. Distinguished from Ladd method, the procedure normalizes the enteromesenteric structural and vascular anatomy by restoring the defective interface between the mesentery and retroperitoneum.^[27]^ Although additional long-term follow-up is required, enteromesenteric corrections have been verified to preclude the risk of midgut volvulus.

In our case, after the diagnosis of midgut malrotation, we gave the patient conservative treatment was provided on the premise of stable vital signs. Nausea and vomiting disappeared after delivery, and no discomfort was found after 1 year of follow-up. Therefore, the time of surgery and termination of pregnancy should be determined according to the situation of the patient and fetus. If their vital signs are stable, surgical treatment may not be required, and the life-threatening complications caused by midgut malrotation should also be paid close attention to and dealt with in time.

Unfortunately, severe hydrocephalus appeared in the fetus at 32 weeks, without abnormalities found in the chromosome single nucleotide polymorphism (SNP) array of the fetus. The complications of drugs administered during hospitalization were investigated, and no studies were found on the relationship between drugs and hydrocephalus. We suspect that long-term electrolyte disorder and acid–base imbalance during pregnancy may lead to fetal hydrocephalus, but the specific mechanism remains to be further studied. It also underscores the significance of early diagnosis and treatment of nausea and vomiting during pregnancy and close examination of the fetus during the following period.

## 5. Conclusion

Symptoms of midgut malrotation may develop during pregnancy and may be nonspecific or vague. A combination of meticulous clinical assessment, short-interval serial observations, and judicious utilization of radiological investigations will facilitate prompt diagnosis and treatment. If a patient’s vital signs are stable, conservative treatment with close monitoring may be a desirable strategy.

## Author contributions

HZ: manuscript writing; LW: manuscript editing; BY: manuscript editing; HS: manuscript editing.
